# Does sleep link child maltreatment to depressive symptoms among incoming first-year college students?

**DOI:** 10.1093/sleepadvances/zpae041

**Published:** 2024-06-19

**Authors:** Darlynn M Rojo-Wissar, Stephanie H Parade, David H Barker, Eliza Van Reen, Katherine M Sharkey, Caroline Gredvig-Ardito, Mary A Carskadon

**Affiliations:** Department of Psychiatry & Human Behavior, Warren Alpert Medical School of Brown University, Providence, RI, USA; Bradley/Hasbro Children’s Research Center, E.P. Bradley Hospital, East Providence, RI, USA; EP Bradley Hospital Sleep Research Laboratory and COBRE Center for Sleep and Circadian Rhythms in Child and Adolescent Mental Health, Providence, RI, USA; Department of Psychiatry & Human Behavior, Warren Alpert Medical School of Brown University, Providence, RI, USA; Bradley/Hasbro Children’s Research Center, E.P. Bradley Hospital, East Providence, RI, USA; Department of Psychiatry & Human Behavior, Warren Alpert Medical School of Brown University, Providence, RI, USA; EP Bradley Hospital Sleep Research Laboratory and COBRE Center for Sleep and Circadian Rhythms in Child and Adolescent Mental Health, Providence, RI, USA; EP Bradley Hospital Sleep Research Laboratory and COBRE Center for Sleep and Circadian Rhythms in Child and Adolescent Mental Health, Providence, RI, USA; Department of Psychiatry & Human Behavior, Warren Alpert Medical School of Brown University, Providence, RI, USA; Department of Medicine, Warren Alpert Medical School of Brown University, Providence, RI, USA; Department of Psychiatry & Human Behavior, Warren Alpert Medical School of Brown University, Providence, RI, USA; EP Bradley Hospital Sleep Research Laboratory and COBRE Center for Sleep and Circadian Rhythms in Child and Adolescent Mental Health, Providence, RI, USA; Department of Psychiatry & Human Behavior, Warren Alpert Medical School of Brown University, Providence, RI, USA; EP Bradley Hospital Sleep Research Laboratory and COBRE Center for Sleep and Circadian Rhythms in Child and Adolescent Mental Health, Providence, RI, USA

## Abstract

**Study Objectives:**

We examined whether sleep (i.e. quality, regularity, and duration) mediated associations between child maltreatment (CM) and depressive symptoms among emerging adults undergoing the major life transition of starting college.

**Methods:**

Students (*N* = 1400; 44% male; 48% non-Hispanic white, 20% non-Hispanic Asian, 15% Hispanic all races, 7% non-Hispanic black, and 10% non-Hispanic other races) completed daily sleep diaries for 9 weeks, followed by the Childhood Trauma Questionnaire-Short Form, Pittsburgh Sleep Quality Index, and the Center for Epidemiologic Studies Depression Scale (CES-D). DSD data were used to compute participants’ Sleep Regularity Index and average 24-hour total sleep time. We used a nonparametric structural equation modeling bootstrap approach and full information maximum likelihood to account for missing data. In model 1, we controlled for sex and race and ethnicity. In model 2, we further adjusted for baseline CES-D scores.

**Results:**

The prevalence of self-reported *moderate-to-severe* CM was 22%. Small but significant indirect effects of CM on greater depressive symptoms through worse sleep quality (*β* = 0.06, 95% CI = 0.04, 0.09) and lower sleep regularity (*β* = 0.02, 95% CI = 0.005, 0.03) were observed in model 1. In model 2, only the indirect effect of sleep quality remained significant (*β* = 0.03, 95% CI = 0.01, 0.06).

**Conclusions:**

Poorer sleep quality may partially account for associations between CM and depressive symptoms during the first semester of college. Including sleep as a target in student health interventions on college campuses may not only help buffer against poor mental health outcomes for students with CM, but also poor academic and socioeconomic outcomes long-term.

**Statement of Significance**

Sleep regularity is understudied in the context of child maltreatment (CM) and related health outcomes. We contribute to filling this gap by showing that CM is associated with poorer sleep quality and more irregular sleep, which in turn are associated with greater depressive symptoms among first-year college students, independent of baseline depressive symptoms. Since seeking mental health care can be stigmatizing, sleep interventions, which can also help improve depressive symptoms, maybe a more accessible approach to promoting well-being and functioning in this group. CM is associated with a lower likelihood of attending college. These associations may be stronger among emerging adults who do not attend college, and in individuals with more diverse sociodemographic backgrounds, which should be examined in future research.

Approximately 33.6% of college students experience depressive symptoms [1], which is significantly higher than the general population. Entering college is a major life transition that comes with significant changes in the environment, social support, responsibilities, identity, and autonomy [2]. While this transition can be an exciting and critical period for personal growth, the accompanying changes and new challenges can also lead to heightened levels of stress and an increased vulnerability to depressive symptoms [3]. Experiences of child maltreatment (CM), including physical, sexual, and emotional abuse and physical and emotional neglect, may further increase this risk [4–6]. Depressive symptoms during college can have a significant negative impact on a student’s academic performance and well-being [7], and in severe cases, lead to drop-out [8]. People with a history of CM are known to have worse educational outcomes and be more likely to drop out of college [9], which may be partially attributable to depressive symptoms. Advancing our understanding of modifiable mechanisms linking CM to elevated depressive symptoms in college students is a key step in developing preventive interventions to reduce CM-related disparities in health and socioeconomic outcomes. Sleep is a promising potential mechanism in need of further exploration.

Behavioral sleep disturbances (e.g. poor sleep quality, high sleep variability, and short or long sleep duration) [10], are a common consequence of CM [11], and are theorized to be one pathway by which CM affects lifelong health, including depression [10, 12]. Preliminary cross-sectional research supports this theory, suggesting that sleep disturbances may partially account for associations between childhood adversity and risk for metabolic syndrome [13], inflammation and hypertension [14], and greater anxiety and depressive symptoms [15]. Most studies, however, have focused on examining links between CM and sleep, and between sleep and health, rather than examining associations among all three. Furthermore, these studies have predominantly focused on sleep quantity and quality, and emerging research suggests that sleep regularity may also play an important role in well-being and academic performance [16–20]. One study found that more irregular sleep was linked to worse mood on average, and day-to-day reductions in irregularity were associated with improvements in the next-day mood [17]. Research examining links between early life adversity and sleep regularity is scarce. In one sample, children with early life adversity reported by their caregivers experienced greater misalignment between weekday and weekend sleep [21]. One reason CM may be associated with more variable sleep patterns is through familial or household chaos or disruption that contributes to failure to develop healthy consistent sleep schedules [22]. Sleep regularity is also highly relevant for college students, whose sleep tends to become more irregular over the transition to college [23].

This study brings together work from the fields of CM and sleep, and from sleep and health to empirically test theoretical models [7, 10, 12] positing that sleep disturbances are a pathway by which CM negatively affects health. We examined whether sleep (i.e. quality, duration, and regularity) during the first semester of college mediates the association between CM and depressive symptoms a few weeks after mid-semester. We hypothesized that poorer sleep would partially account for associations between CM and depressive symptoms among the community sample of students.

## Materials and Methods

### Participants and procedure

Data were derived from a larger study of matriculating college students at a private university in the Northeastern United States recruited from 2009 to 2014 [24, 25]. Students who participated in a one-time survey in the months before college were recruited to attend an initial visit at the university to review study procedures and provide written informed consent (*N* = 1400 enrolled). Participants then received a daily email with a link to their electronic daily sleep diaries (DSDs), which they completed for 9 weeks, starting from the first day of class. Near completion of the final DSDs of the semester, participants were provided with a link to the final outcome survey, which included assessment of CM [26], global sleep quality, and depressive symptoms [27]. Note that due to a programming error in 2010–2012, the CM measure was not included in the outcome survey. In those cases, students completed the CM measure via a separate link to an online secure site at the end of 2012. The proportion of participants who completed the CM measure separately in this sample was 49%. The Lifespan Institutional Review Board approved all study procedures.

### Measures

#### Sleep.

The first item in the DSD asked whether participants had slept in the last 24 hours. If they responded no, sleep duration was coded as 0 hours. If they responded yes, they were asked “For your main sleep period in the last 24 hours”: “How long did you sleep?” “What time did you try to fall asleep?” “What time did you finally wake up?” “Estimate how many minutes it took you to fall asleep.” “Estimate how many minutes you were awake after you fell asleep.” The Sleep Regularity Index (SRI) [28] was computed using these data, with higher scores indicating more regular sleep patterns. The SRI algorithm implicitly imputes the overall SRI mean for missing diary days. Because the timing of naps and night awakenings were not collected, each major wake or sleep episode was weighted by the proportion of time spent in the opposite state [29]. This approach may underestimate regularity because it presumes that naps or awakenings do not occur at the same time one day to the next. Average 24-hour total sleep time in hours was computed as the average daily duration of the time in bed interval (from the time participants report trying to fall asleep to the time they finally woke up in the morning) minus sleep onset latency and wake after-sleep onset, plus nap duration. The Pittsburgh Sleep Quality Index (PSQI) [30] was used to assess participant-reported global sleep quality over the past month. Higher scores indicate worse sleep quality and a score of > 5 indicates poor sleep quality.

#### Child maltreatment.

The 28-item Childhood Trauma Questionnaire-short form (CTQ-SF) [26] was used to retrospectively measure participant-reported childhood experiences of physical, sexual, and emotional abuse, and physical and emotional neglect. Response options were on a 5-point Likert scale ranging from “never true” (1) to “very often true” (5). A CTQ-SF total score was computed (internal consistency reliability: α = 0.87), with higher scores indicating more severe maltreatment. Subscale scores were also computed. The CTQ-SF has been validated across a range of populations [26], including college students [31].

#### Depressive Symptoms.

The 20-item Center for Epidemiologic Studies—Depression Scale (CES-D) [27] was used to measure depressive symptoms during the past week, such as feeling depressed, hopelessness, and poor appetite. The CES-D has been validated in young adults and is considered acceptable and reliable in college students [32]. A total score was computed (internal consistency reliability: α = 0.90), with higher scores indicating greater depressive symptoms.

#### Other Covariates.

The university provided participant sex (male/female) and country of residence (coded as resident status: United States vs. not). Participants reported on their race and ethnicity (categorized as non-Hispanic white, non-Hispanic Asian, non-Hispanic black, Hispanic of all races, and others).

### Data analysis

**Figure 1.**
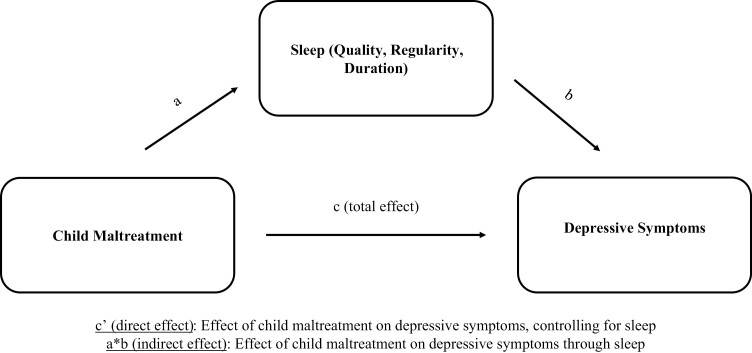
Hypothesized mediation model of child maltreatment on depressive symptoms through sleep with estimated paths and effects.

We used bootstrapped structural equation modeling (1000 bootstrap samples) with percentile-based 95% confidence intervals [33] to examine whether sleep-mediated effects of CM on depressive symptoms. We estimated several effects relevant to mediation analysis, including the effect of CM on sleep (a in Figure 1), sleep on depressive symptoms (b), CM on depressive symptoms through sleep (indirect effect, a*b), CM on depressive symptoms keeping sleep constant (direct effect, c’), and the total effect (c) which is the combination of the indirect and direct effects (or the effect of CM on depressive symptoms). All models controlled for sex and race and ethnicity for both the mediator and outcome [15], consistent with previous research, and the outcome was further adjusted using baseline depressive symptoms. International student status, was not associated with differences in CM or sleep, thus we did not control for it. Significant indirect effects indicated mediation. Full information maximum likelihood was used to account for missing data. Analyses were completed in Stata version 18.0 (StataCorp, College Station, TX).

## Results

Descriptive statistics are reported in Table 1. The mean age of the participants was 18.65 years (SD = 0.50), 56.4% were female, 47.8% were non-Hispanic white, and 21.5% endorsed moderate to severe CM (Additional CM descriptive information is reported in Supplementary Table 1).

**Table 1.** Participant Characteristics (*N* = 1400)

	Mean (± SD) or *n* (%)	Range	*n*
Age at the Start of University	18.65 (0.50)	17.52, 21.97	1400
Sex			1400
Male	610 (43.6%)		
Female	790 (56.4%)		
Race and Ethnicity			1383
NH white	661 (47.8%)		
NH Asian	282 (20.4%)		
NH black	91 (6.6%)		
Hispanic all races	207 (15.0%)		
Other	142 (10.3%)		
US resident status			1399
Non-US resident	202 (14.4%)		
US resident	1197 (85.6%)		
CTQ-SF total score	33.27 (9.02)	25, 101	855
Baseline CES-D scale score	11.90 (8.86)	0, 52	1391
Sleep Regularity Index	74.55 (7.93)	41.11, 94.58	998
24-hour sleep duration, hours	7.46 (0.62)	3.89, 9.42	998
Global Pittsburgh Sleep Quality Index Score	4.71 (2.11)	0, 16	979
CES-D scale score	14.83 (9.69)	0, 51	982

NH, non-Hispanic; CES-D, Center for Epidemiologic Studies Depression Scale; CTQ-SF, Childhood Trauma Questionnaire- Short Form. For the sleep regularity index and 24-hour sleep duration, Ns indicate the number of participants who completed ≥ 66% of their daily sleep diaries (71%). Possible ranges: CTQ-SF, 5–125; PSQI, 0–21; and CES-D, 0–60.

In model 1, both direct effects and indirect effects of CM on greater depressive symptoms through lower sleep regularity and poorer sleep quality were observed (Table 2). In model 2, the only indirect effect that remained significant was sleep quality. Supplemental analyses examining CM subtypes are reported in Supplementary Tables 2:4.

**Table 2.** Mediation Effects of Hypothesized Models of Child Maltreatment (CTQ-SF) on Depressive Symptoms (CES-D) Through Sleep

	Model 1 (adjusted for sex and race and ethnicity)					Model 2 (further adjusted for depressive symptoms)				
Path	B	SE	*β*	95% CI LL	95% CI UL	B	SE	*β*	95% CI LL	95% CI UL
*Pittsburgh Sleep Quality Index*										
a	*0.04*	*0.01*	*0.17*	*0.10*	*0.24*	*0.03*	*0.01*	*0.12*	*0.04*	*0.19*
b	*1.71*	*0.14*	*0.37*	*0.31*	*0.43*	*1.29*	*0.14*	*0.28*	*0.22*	*0.34*
a × b (indirect effect)	*0.07*	*0.02*	*0.06*	*0.04*	*0.09*	*0.04*	*0.01*	*0.03*	*0.01*	*0.06*
c (total effect)	*0.31*	*0.05*	*0.28*	*0.21*	*0.37*	*0.19*	*0.05*	*0.18*	*0.10*	*0.26*
c’ (direct effect)	*0.24*	*0.05*	*0.22*	*0.14*	*0.31*	*0.16*	*0.05*	*0.15*	*0.07*	*0.23*
*Sleep Regularity Index*										
a	*−0.12*	*0.03*	*−0.14*	*−0.21*	*−0.07*	*−0.10*	*0.03*	*−0.12*	*−0.19*	*−0.05*
b	*−0.14*	*0.04*	*−0.11*	*−0.19*	*−0.04*	*−0.09*	*0.04*	*−0.07*	*−0.13*	*−0.01*
a × b (indirect effect)	*0.02*	*0.01*	*0.02*	*0.005*	*0.03*	*0.01*	*0.01*	*0.01*	*0.00*	*0.02*
c (total effect)	*0.31*	*0.05*	*0.28*	*0.21*	*0.37*	*0.19*	*0.04*	*0.18*	*0.10*	*0.26*
c’ (direct effect)	*0.29*	*0.05*	*0.27*	*0.19*	*0.35*	*0.18*	*0.04*	*0.17*	*0.10*	*0.25*
*24-hour average sleep duration*										
a	−0.001	0.002	−0.01	−0.08	0.06	0.001	0.002	0.01	−0.06	0.08
b	−0.99	0.54	−0.06	−0.13	0.004	−0.46	0.48	−0.03	−0.09	0.03
a × b (indirect effect)	0.001	0.003	0.001	−0.004	0.01	−0.0003	0.002	−0.0003	−0.004	0.003
c (total effect)	*0.31*	*0.05*	*0.29*	*0.21*	*0.37*	*0.19*	*0.04*	*0.18*	*0.11*	*0.26*
c’ (direct effect)	*0.31*	*0.05*	*0.29*	*0.21*	*0.37*	*0.20*	*0.04*	*0.18*	*0.11*	*0.27*

SE, standard error; CI, confidence interval; LL, lower limit; UL, upper limit. Significant effects are in italics. 95% CIs are percentile-based. B and SE are unstandardized, β and 95% CIs are standardized. The Sleep Regularity Index and 24-hour average sleep duration are computed from the daily sleep diary data.

## Discussion

The present study aimed to investigate the mediating role of sleep during the first ~9 weeks of college in the association between CM and depressive symptoms a few weeks after mid-semester among a community sample of college students. When accounting for baseline depressive symptoms, poorer sleep quality accounted for a small but significant portion of the association between CM and later depressive symptoms. Though CM was associated with lower sleep regularity, which was in turn associated with greater depressive symptoms, the indirect effect was not significant in models adjusting for baseline depressive symptoms. Our findings align with theoretical models suggesting that sleep disturbances are a mechanism by which CM negatively affects health [7, 10, 12], and suggest that subjective sleep quality may be the most relevant aspect of sleep in this framework when depressive symptoms are the outcome. This is consistent with recent reviews demonstrating that CM has the strongest and most robust associations with subjective sleep quality versus other aspects of sleep [11, 34].

Our study extends the literature by providing some empirical evidence for a mediating role of subjective sleep quality in the CM-depressive symptoms relationship among first-year college students, and no such role of sleep duration. Our findings also suggest that CM is associated with more irregular sleep, which is in turn associated with greater depressive symptoms. Investigation of sleep regularity as the sleep variable of interest is novel, as most studies have focused on sleep quantity and quality. Sleep regularity is also highly relevant for first-year college students who experience a major shift toward greater autonomy around their own schedules, coupled with increased academic demands and social opportunities [2]. Measuring sleep patterns during participants’ first semester of college allowed us to assess how students responded to a “sleep challenge,” and whether CM played a role in their adjustment. Of note, associations among CM, sleep, and depressive symptoms were smaller than anticipated, which may be partially attributable to the lower rates of CM reported in our sample compared to those in previous studies [15, 35].

The present study is strengthened by the timing of assessments, which allowed us to examine how retrospectively reported CM prior to college was related to sleep, measured by DSDs over the first ~9 weeks of the semester (And by the PSQI), and in turn, depressive symptoms after mid-semester. Previous studies examining sleep as a potential mediator between CM and health-related outcomes have largely used measures of CM, sleep, and health collected at the same timepoint [13–15]. Furthermore, we controlled for the effects of baseline depressive symptoms. Last, the use of a sample of emerging adult college students is advantageous as this developmental period is considered a time of increased risk for both sleep disturbances and depressive symptoms, and the campus environment provides unique opportunities for prevention and intervention efforts. There are also limitations of this research. We did not measure socioeconomic status or other potential psychosocial risk or protective factors like parental or peer relationships, which could affect associations among CM, sleep, and depressive symptoms and limit the generalizability of this study. The CTQ-SF is retrospective report and may be subject to related biases (e.g. recall), and last, all measures were based on self-report. Future research in this area should also include objective assessment of sleep, and more diverse samples with greater CM exposure.

Findings from this study highlight the importance of subjective sleep quality as a potential mechanism through which CM influences mental health outcomes in college students, particularly depressive symptoms. Addressing sleep in student health interventions may help mitigate the risk of poor mental health outcomes and improve academic and socioeconomic outcomes, particularly for students with a history of CM.

## Supplementary Material

zpae041_suppl_Supplementary_Tables_S1-S4

## Data Availability

The data underlying this article cannot be shared publicly to maintain the privacy of the individuals who participated in the study. The data may be shared upon reasonable request to the corresponding author.
