# Supplementary material for: Does sleep link child maltreatment to depressive symptoms among incoming first-year college students?
Source: Sleep Adv. 2024 Jun 19;5(1):zpae041. doi: 10.1093/sleepadvances/zpae041 (PMC11229310; doi:10.1093/sleepadvances/zpae041)
Supplement: zpae041_suppl_Supplementary_Tables_S1-S4 [file zpae041_suppl_supplementary_tables_s1-s4.docx]

**Title Page**

**Title:** Does Sleep Link Child Maltreatment to Depressive Symptoms among Incoming First-Year College Students?

**Authors and Institutions:** Darlynn M. Rojo-Wissar^a,b,c^, Stephanie H. Parade^a,b^, David H. Barker^a,c^, Eliza Van Reen^c^, Katherine M. Sharkey^a,d^, Caroline Gredvig-Ardito^a,c^, & Mary A. Carskadon^a,c^

^a^Department of Psychiatry & Human Behavior, Warren Alpert Medical School of Brown University, Providence, RI, USA; ^b^Bradley/Hasbro Children’s Research Center, E.P. Bradley Hospital, East Providence, RI, USA; ^c^ EP Bradley Hospital Sleep Research Laboratory and COBRE Center for Sleep and Circadian Rhythms in Child and Adolescent Mental Health, Providence, RI, USA; ^d^Department of Medicine, Warren Alpert Medical School of Brown University, Providence, RI, USA

**Corresponding Author:**

Darlynn M. Rojo-Wissar

Bradley/Hasbro Children’s Research Center, E. P. Bradley Hospital

Department of Psychiatry and Human Behavior, Warren Alpert Medical School of Brown University,

1011 Veterans Memorial Parkway

East Providence, RI 02915

[darlynn_rojo-wissar@brown.edu](mailto:darlynn_rojo-wissar@brown.edu)

| **Supplemental Table 1** |  |  |
| --- | --- | --- |
| Childhood Trauma Questionnaire-Short Form Supplemental Information | | |
|  | **n (%)** | **n** |
| Any Moderate to Severe Maltreatment |  |  |
| No | 671 (78.5%) | 855 |
| Yes | 184 (21.5%) |  |
| Number of Moderate to Severe Maltreatment Types |  | 855 |
| 0 | 671 (78.5%) |  |
| 1 | 121 (14.2%) |  |
| 2 | 40 (4.7%) |  |
| 3 | 13 (1.5%) |  |
| 4 | 5 (0.6%) |  |
| 5 | 5 (0.6%) |  |
| Moderate to Severe Emotional Abuse |  | 854 |
| No | 806 (94.4%) |  |
| Yes | 48 (5.6%) |  |
| Moderate to Severe Physical Abuse |  | 855 |
| No | 823 (96.3%) |  |
| Yes | 32 (3.7%) |  |
| Moderate to Severe Sexual Abuse |  | 854 |
| No | 821 (96.1%) |  |
| Yes | 33 (3.9%) |  |
| Moderate to Severe Emotional Neglect |  | 854 |
| No | 784 (91.8%) |  |
| Yes | 70 (8.2%) |  |
| Moderate to Severe Physical Neglect |  | 855 |
| No | 753 (88.1%) |  |
| Yes | 102 (11.9%) |  |
|  |  |  |
| Emotional Abuse Subscale Score (Range: 5, 24) | 7.01 (2.91) | 854 |
| Physical Abuse Subscale Score (Range: 5, 20) | 5.67 (1.71) | 855 |
| Sexual Abuse Subscale Score (Range: 5, 25) | 5.43 (2.16) | 854 |
| Emotional Neglect Subscale Score (Range: 5, 23) | 8.38 (3.60) | 854 |
| Physical Neglect Subscale Score (Range: 5, 20) | 6.80 (2.33) | 855 |
| *Note*. Internal consistency reliability for each scale in our sample was as follows: physical abuse α = .69, sexual abuse α = .93, emotional abuse α = .79, physical neglect α = .46, emotional neglect α = .84. Moderate to severe maltreatment cutoff scores for each subscale are emotional abuse ≥13, physical abuse ≥10, sexual abuse ≥8, emotional neglect ≥15, and physical neglect ≥10. | | |

| **Supplemental Table 2** | |  |  |  |  |  |  |  |  |  |
| --- | --- | --- | --- | --- | --- | --- | --- | --- | --- | --- |
| Mediation Effects of Hypothesized Models of Child Maltreatment Types (Childhood Trauma Questionnaire Short Form Subscales) on Depressive Symptoms Through Sleep Quality (Global Pittsburgh Sleep Quality Index Score) | | | | | | | | | | |
|  | **Model 1**  **(adjusted for sex and race and ethnicity)** | | | | | **Model 2**  **(further adjusted for depressive symptoms)** | | | | |
| Path | B | SE | β | 95% CI LL | 95% CI UL | B | SE | β | 95% CI LL | 95% CI UL |
| ***Emotional Abuse*** |  |  |  |  |  |  |  |  |  |  |
| a | **0.11** | **0.03** | **0.15** | **0.07** | **0.22** | **0.07** | **0.03** | **0.10** | **0.02** | **0.17** |
| b | **1.75** | **0.14** | **0.38** | **0.32** | **0.44** | **1.32** | **0.14** | **0.29** | **0.23** | **0.34** |
| a x b (indirect effect) | **0.19** | **0.05** | **0.06** | **0.03** | **0.09** | **0.09** | **0.04** | **0.03** | **0.01** | **0.05** |
| c (total effect) | **0.81** | **0.14** | **0.24** | **0.16** | **0.33** | **0.47** | **0.13** | **0.14** | **0.07** | **0.22** |
| c' (direct effect) | **0.62** | **0.14** | **0.19** | **0.11** | **0.27** | **0.38** | **0.13** | **0.11** | **0.04** | **0.19** |
| ***Physical Abuse*** |  |  |  |  |  |  |  |  |  |  |
| a | **0.11** | **0.04** | **0.09** | **0.02** | **0.16** | **0.09** | **0.04** | **0.08** | **0.002** | **0.14** |
| b | **1.86** | **0.14** | **0.40** | **0.34** | **0.46** | **1.36** | **0.14** | **0.30** | **0.24** | **0.36** |
| a x b (indirect effect) | **0.21** | **0.08** | **0.04** | **0.01** | **0.06** | **0.13** | **0.06** | **0.02** | **0.001** | **0.04** |
| c (total effect) | 0.39 | 0.29 | 0.07 | -0.03 | 0.18 | 0.21 | 0.27 | 0.04 | -0.05 | 0.13 |
| c' (direct effect) | 0.18 | 0.30 | 0.03 | -0.07 | 0.14 | 0.08 | 0.27 | 0.01 | -0.07 | 0.11 |
| ***Sexual Abuse*** |  |  |  |  |  |  |  |  |  |  |
| a | -0.03 | 0.04 | -0.03 | -0.11 | 0.05 | -0.04 | 0.04 | -0.04 | -0.11 | 0.04 |
| b | **1.88** | **0.14** | **0.41** | **0.35** | **0.46** | **1.38** | **0.14** | **0.30** | **0.24** | **0.36** |
| a x b (indirect effect) | -0.06 | 0.07 | -0.01 | -0.05 | 0.02 | -0.05 | 0.05 | -0.01 | -0.03 | 0.01 |
| c (total effect) | 0.33 | 0.25 | 0.07 | -0.03 | 0.18 | 0.30 | 0.22 | 0.07 | -0.03 | 0.16 |
| c' (direct effect) | 0.39 | 0.22 | 0.09 | -0.01 | 0.19 | 0.35 | 0.20 | 0.08 | -0.01 | 0.16 |
| ***Emotional Neglect*** |  |  |  |  |  |  |  |  |  |  |
| a | **0.10** | **0.02** | **0.17** | **0.10** | **0.24** | **0.07** | **0.02** | **0.12** | **0.05** | **0.19** |
| b | **1.71** | **0.14** | **0.37** | **0.31** | **0.43** | **1.30** | **0.14** | **0.28** | **0.23** | **0.34** |
| a x b (indirect effect) | **0.17** | **0.04** | **0.06** | **0.04** | **0.09** | **0.09** | **0.03** | **0.03** | **0.01** | **0.05** |
| c (total effect) | **0.73** | **0.11** | **0.27** | **0.20** | **0.35** | **0.44** | **0.10** | **0.16** | **0.10** | **0.23** |
| c' (direct effect) | **0.56** | **0.10** | **0.21** | **0.14** | **0.28** | **0.35** | **0.09** | **0.13** | **0.07** | **0.20** |
| ***Physical Neglect*** |  |  |  |  |  |  |  |  |  |  |
| a | **0.13** | **0.03** | **0.15** | **0.08** | **0.21** | **0.10** | **0.03** | **0.11** | **0.04** | **0.17** |
| b | **1.76** | **0.14** | **0.38** | **0.32** | **0.44** | **1.31** | **0.14** | **0.28** | **0.22** | **0.34** |
| a x b (indirect effect) | **0.23** | **0.06** | **0.06** | **0.03** | **0.08** | **0.13** | **0.04** | **0.03** | **0.01** | **0.05** |
| c (total effect) | **0.94** | **0.19** | **0.23** | **0.14** | **0.31** | **0.64** | **0.16** | **0.15** | **0.08** | **0.23** |
| c' (direct effect) | **0.71** | **0.18** | **0.17** | **0.09** | **0.25** | **0.51** | **0.16** | **0.12** | **0.05** | **0.20** |
| *Note*. SE = standard error, CI = confidence interval, LL = lower limit, UL = upper limit. Significant effects are bolded. 95% CIs are percentile based. B and SE are unstandardized, β and 95% CIs are standardized. | | | | | | | | | | |

| **Supplemental Table 3** | |  |  |  |  |  |  |  |  |  |
| --- | --- | --- | --- | --- | --- | --- | --- | --- | --- | --- |
| Mediation Effects of Hypothesized Models of Child Maltreatment Types (Childhood Trauma Questionnaire Short Form Subscales) on Depressive Symptoms Through Sleep Regularity (Daily Diary Derived Sleep Regularity Index) | | | | | | | | | | |
|  | **Model 1**  **(adjusted for sex and race and ethnicity)** | | | | | **Model 2**  **(further adjusted for depressive symptoms)** | | | | |
| Path | B | SE | β | 95% CI LL | 95% CI UL | B | SE | β | 95% CI LL | 95% CI UL |
| ***Emotional Abuse*** |  |  |  |  |  |  |  |  |  |  |
| a | **-0.32** | **0.11** | **-0.12** | **-0.20** | **-0.05** | **-0.27** | **0.11** | **-0.10** | **-0.18** | **-0.03** |
| b | **-0.15** | **0.04** | **-0.12** | **-0.19** | **-0.05** | **-0.09** | **0.04** | **-0.08** | **-0.14** | **-0.01** |
| a x b (indirect effect) | **0.05** | **0.02** | **0.01** | **0.004** | **0.03** | 0.03 | 0.02 | 0.01 | 0.00 | 0.02 |
| c (total effect) | **0.81** | **0.14** | **0.24** | **0.17** | **0.33** | **0.47** | **0.13** | **0.14** | **0.07** | **0.22** |
| c' (direct effect) | **0.76** | **0.14** | **0.23** | **0.15** | **0.31** | **0.44** | **0.13** | **0.13** | **0.06** | **0.21** |
| ***Physical Abuse*** |  |  |  |  |  |  |  |  |  |  |
| a | **-0.37** | **0.18** | **-0.08** | **-0.15** | **-0.02** | **-0.33** | **0.17** | **-0.07** | **-0.14** | **-0.01** |
| b | **-0.17** | **0.05** | **-0.14** | **-0.22** | **-0.07** | **-0.11** | **0.04** | **-0.09** | **-0.15** | **-0.02** |
| a x b (indirect effect) | 0.06 | 0.04 | 0.01 | 0.00 | 0.02 | 0.04 | 0.02 | 0.01 | 0.00 | 0.02 |
| c (total effect) | 0.38 | 0.29 | 0.07 | -0.03 | 0.17 | 0.21 | 0.27 | 0.04 | -0.05 | 0.13 |
| c' (direct effect) | 0.32 | 0.29 | 0.06 | -0.04 | 0.16 | 0.17 | 0.26 | 0.03 | -0.05 | 0.12 |
| ***Sexual Abuse*** |  |  |  |  |  |  |  |  |  |  |
| a | **-0.36** | **0.10** | **-0.10** | **-0.15** | **-0.04** | **-0.35** | **0.10** | **-0.10** | **-0.15** | **-0.04** |
| b | **-0.17** | **0.05** | **-0.14** | **-0.22** | **-0.07** | **-0.10** | **0.04** | **-0.08** | **-0.15** | **-0.02** |
| a x b (indirect effect) | **0.06** | **0.02** | **0.01** | **0.01** | **0.03** | **0.04** | **0.02** | **0.01** | **0.002** | **0.02** |
| c (total effect) | 0.33 | 0.25 | 0.07 | -0.04 | 0.18 | 0.29 | 0.22 | 0.06 | -0.03 | 0.16 |
| c' (direct effect) | 0.26 | 0.25 | 0.06 | -0.05 | 0.17 | 0.25 | 0.22 | 0.06 | -0.04 | 0.15 |
| ***Emotional Neglect*** |  |  |  |  |  |  |  |  |  |  |
| a | **-0.24** | **0.08** | **-0.11** | **-0.18** | **-0.04** | **-0.19** | **0.08** | **-0.09** | **-0.16** | **-0.02** |
| b | **-0.15** | **0.04** | **-0.12** | **-0.19** | **-0.05** | **-0.09** | **0.04** | **-0.08** | **-0.14** | **-0.01** |
| a x b (indirect effect) | **0.04** | **0.02** | **0.01** | **0.003** | **0.03** | 0.02 | 0.01 | 0.01 | 0.00 | 0.02 |
| c (total effect) | **0.74** | **0.11** | **0.27** | **0.20** | **0.35** | **0.44** | **0.10** | **0.16** | **0.10** | **0.23** |
| c' (direct effect) | **0.70** | **0.11** | **0.26** | **0.19** | **0.34** | **0.42** | **0.10** | **0.16** | **0.09** | **0.23** |
| ***Physical Neglect*** |  |  |  |  |  |  |  |  |  |  |
| a | -0.19 | 0.13 | -0.05 | -0.13 | 0.02 | -0.13 | 0.13 | -0.04 | -0.11 | 0.03 |
| b | **-0.16** | **0.04** | **-0.13** | **-0.21** | **-0.07** | **-0.10** | **0.04** | **-0.08** | **-0.15** | **-0.02** |
| a x b (indirect effect) | 0.03 | 0.02 | 0.01 | -0.002 | 0.02 | 0.01 | 0.01 | 0.003 | -0.003 | 0.01 |
| c (total effect) | **0.94** | **0.18** | **0.23** | **0.14** | **0.31** | **0.64** | **0.16** | **0.15** | **0.08** | **0.23** |
| c' (direct effect) | **0.91** | **0.18** | **0.22** | **0.14** | **0.30** | **0.63** | **0.16** | **0.15** | **0.08** | **0.22** |
| *Note*. SE = standard error, CI = confidence interval, LL = lower limit, UL = upper limit. Significant effects are bolded. 95% CIs are percentile based. B and SE are unstandardized, β and 95% CIs are standardized. | | | | | | | | | | |

| **Supplemental Table 4** | |  |  |  |  |  |  |  |  |  |
| --- | --- | --- | --- | --- | --- | --- | --- | --- | --- | --- |
| Mediation effects of Hypothesized Models of Child Maltreatment Types (Childhood Trauma Questionnaire Short Form Subscales) on Depressive Symptoms Through Sleep Duration | | | | | | | | | | |
|  | **Model 1**  **(adjusted for sex and race and ethnicity)** | | | | | **Model 2**  **(further adjusted for depressive symptoms)** | | | | |
| Path | B | SE | β | 95% CI LL | 95% CI UL | B | SE | β | 95% CI LL | 95% CI UL |
| ***Emotional Abuse*** |  |  |  |  |  |  |  |  |  |  |
| a | -0.01 | 0.01 | -0.03 | -0.10 | 0.05 | -0.002 | 0.01 | -0.01 | -0.08 | 0.06 |
| b | -0.92 | 0.55 | -0.06 | -0.13 | 0.01 | -0.41 | 0.49 | -0.03 | -0.09 | 0.03 |
| a x b (indirect effect) | 0.01 | 0.01 | 0.002 | -0.003 | 0.008 | 0.001 | 0.01 | 0.0002 | -0.003 | 0.004 |
| c (total effect) | **0.81** | **0.14** | **0.24** | **0.17** | **0.33** | **0.47** | **0.13** | **0.14** | **0.07** | **0.22** |
| c' (direct effect) | **0.81** | **0.14** | **0.24** | **0.16** | **0.33** | **0.47** | **0.13** | **0.14** | **0.07** | **0.22** |
| ***Physical Abuse*** |  |  |  |  |  |  |  |  |  |  |
| a | 0.001 | 0.01 | 0.004 | -0.06 | 0.071 | 0.004 | 0.01 | 0.01 | -0.05 | 0.08 |
| b | -1.01 | 0.56 | -0.07 | -0.14 | 0.01 | -0.43 | 0.49 | -0.03 | -0.09 | 0.03 |
| a x b (indirect effect) | -0.001 | 0.01 | -0.0003 | -0.01 | 0.005 | -0.002 | 0.01 | -0.0003 | -0.003 | 0.002 |
| c (total effect) | 0.40 | 0.29 | 0.07 | -0.03 | 0.18 | 0.22 | 0.27 | 0.04 | -0.05 | 0.13 |
| c' (direct effect) | 0.40 | 0.29 | 0.07 | -0.03 | 0.18 | 0.22 | 0.27 | 0.04 | -0.05 | 0.13 |
| ***Sexual Abuse*** |  |  |  |  |  |  |  |  |  |  |
| a | 0.01 | 0.01 | 0.03 | -0.04 | 0.09 | 0.01 | 0.01 | 0.03 | -0.04 | 0.09 |
| b | -1.07 | 0.56 | -0.07 | -0.14 | 0.002 | -0.48 | 0.49 | -0.03 | -0.09 | 0.03 |
| a x b (indirect effect) | -0.01 | 0.01 | -0.002 | -0.01 | 0.003 | -0.004 | 0.01 | -0.001 | -0.005 | 0.002 |
| c (total effect) | 0.34 | 0.25 | 0.08 | -0.03 | 0.18 | 0.30 | 0.22 | 0.07 | -0.03 | 0.16 |
| c' (direct effect) | 0.35 | 0.25 | 0.08 | -0.03 | 0.18 | 0.30 | 0.22 | 0.07 | -0.03 | 0.16 |
| ***Emotional Neglect*** |  |  |  |  |  |  |  |  |  |  |
| a | -0.01 | 0.01 | -0.05 | -0.124 | 0.02 | -0.01 | 0.01 | -0.03 | -0.11 | 0.04 |
| b | -0.80 | 0.54 | -0.05 | -0.12 | 0.02 | -0.35 | 0.48 | -0.02 | -0.08 | 0.04 |
| a x b (indirect effect) | 0.01 | 0.01 | 0.003 | -0.002 | 0.01 | 0.002 | 0.005 | 0.001 | -0.002 | 0.006 |
| c (total effect) | **0.74** | **0.11** | **0.27** | **0.20** | **0.36** | **0.44** | **0.10** | **0.16** | **0.10** | **0.23** |
| c' (direct effect) | **0.73** | **0.11** | **0.27** | **0.20** | **0.35** | **0.44** | **0.10** | **0.16** | **0.10** | **0.23** |
| ***Physical Neglect*** |  |  |  |  |  |  |  |  |  |  |
| a | 0.01 | 0.01 | 0.05 | -0.01 | 0.11 | 0.02 | 0.01 | 0.063 | -0.001 | 0.13 |
| b | **-1.23** | **0.56** | **-0.08** | **-0.15** | **-0.01** | -0.61 | 0.49 | -0.04 | -0.10 | 0.02 |
| a x b (indirect effect) | -0.02 | 0.01 | -0.004 | -0.01 | 0.001 | -0.01 | 0.01 | -0.002 | -0.01 | 0.001 |
| c (total effect) | **0.95** | **0.18** | **0.23** | **0.14** | **0.31** | **0.65** | **0.16** | **0.16** | **0.08** | **0.23** |
| c' (direct effect) | **0.97** | **0.19** | **0.23** | **0.15** | **0.32** | **0.66** | **0.16** | **0.16** | **0.08** | **0.23** |

*Note*. SE = standard error, CI = confidence interval, LL = lower limit, UL = upper limit. Significant effects are bolded. 95% CIs are percentile based. B and SE are unstandardized, β and 95% CIs are standardized.
